# Subtypes of Type 2 Diabetes and Prediabetes: Mortality and Excess Life Lost in South Asians

**DOI:** 10.21203/rs.3.rs-6819284/v1

**Published:** 2025-06-23

**Authors:** Ram Jagannathan, Dimple Kondal, Pradeep Tiwari, Mohan Deepa, Unjali Gujral, Shivani Patel, Sailesh Mohan, Ranjit Anjana, Lisa Staimez, Ruby Gupta, Yara Beyh, Ayodipupo Oguntade, Howard Chang, Mohammed Ali, Arshed Quyyumi, Yan Sun, Dorairaj Prabhakaran, Nikhil Tandon, Viswanathan Mohan, KM Venkat Narayan

**Affiliations:** Emory Global Diabetes Research Center, Woodruff Health Sciences Center and Emory University; Centers for Chronic Disease Control, India; Emory University; 7. Madras Diabetes Research Foundation, Chennai, India.; Emory Global Diabetes Research Center, Woodruff Health Sciences Center and Emory University; Emory University; Public Health Foundation of India; Madras Diabetes Research Foundation & Dr. Mohan’s Diabetes Specialities Centre; Emory Global Diabetes Research Center, Woodruff Health Sciences Center and Emory University; Centre for Chronic Disease Control (CCDC); Emory Global Diabetes Research Center, Woodruff Health Sciences Center and Emory University; Emory Global Diabetes Research Center, Woodruff Health Sciences Center and Emory University; Emory University; Emory University; Emory University; Emory University; Executive Director, Centre for Chronic Disease Control and Distinguished Professor of Public Health, Public Health Foundation of India; All India Institute of Medical Sciences (AIIMS); Madras Diabetes Research Foundation & Dr. Mohan’s Diabetes Specialities Centre; 5. Hubert Department of Global Health, Emory University Rollins School of Public Health, Atlanta, GA

## Abstract

**Background:**

Current definitions of type 2 diabetes (T2D) and prediabetes do not capture their pathophysiological heterogeneity. We investigated data-driven subtypes of T2D and prediabetes and evaluated their associations with mortality.

**Methods:**

We analyzed data from 14,306 South Asian participants from the CArdiometabolic Risk Reduction cohort using unsupervised k-means clustering based on five variables: age, BMI, HbA1c, insulin resistance, and beta-cell dysfunction. For each subtype of T2D or prediabetes, we estimated Cox hazard ratios (HRs) for all-cause and cardiovascular disease (CVD) mortality and excess years of life lost compared to normal glucose tolerance.

**Results:**

Among 2,639 participants with T2D, three subtypes emerged: Severe Insulin-Deficient Diabetes (SIDD;23.0%), Mild Insulin-Deficient Diabetes (MIDD;54.5%), and Severe Insulin-Resistant Diabetes (SIRD;22.5%). Among 4,992 participants with prediabetes, two subtypes were identified: Insulin-Deficient Prediabetes (IDPD;66.0%) and Insulin-Resistant Prediabetes (IRPD;34.0%). Over a median follow-up of 10.6 years, 1,076 deaths occurred (405 due to CVD). Compared with normal glucose tolerance, SIDD had the highest all-cause mortality HR (3.34 [95%CI, 2.39 to 4.68]), followed by MIDD (1.39[95%CI, 1.05 to 1.84]) and SIRD (1.67[95%CI, 1.15 to 2.41]). Among prediabetes subtypes, IDPD was associated with increased all-cause (HR: 1.32 [95%CI, 1.03 to 1.68]) and CVD mortality (HR:1.53 [95%CI, 1.00 to 2.34]), whereas IRPD was not. Excess years of life lost were greatest for SIDD (17.7 years), followed by MIDD (12.8 years) and SIRD (12.0 years).

**Conclusions:**

Insulin-deficient subtypes made up a high proportion of T2D and prediabetes cases, harboring increased mortality hazards and excess years of life lost relative to normal glucose tolerance.

## Introduction

Globally, an estimated 828 million adults were living with diabetes in 2022, with 90–95% of cases being type 2 diabetes.^1^ An additional 762 million adults were estimated to have prediabetes in 2021.^2^ Both type 2 diabetes and prediabetes are well-established risk factors for all-cause and cardiovascular disease mortality, contributing substantially to premature death.^3–5^ Yet, categorizing type 2 diabetes and prediabetes as monolithic groups based solely on fixed glycemic thresholds overlooks their complex pathophysiology, therapeutic responses, and prognoses that make these conditions quite heterogeneous.^6^

Recent advances in data-driven clustering analyses have identified novel subclassifications of type 2 diabetes,^7–13^ consistently replicated in over 20 studies spanning diverse ancestries.^14^ These analyses highlight etiological distinctions in type 2 diabetes pathophysiology across populations: while obesity and insulin resistance, compounded by age-related metabolic aberrations, predominate in type 2 diabetes subtypes among Scandinavian and Northern European populations, South Asians more frequently exhibit primary deficits in insulin secretion.^8–13,15^ Furthermore, evidence from predominantly European cohorts suggests that type 2 diabetes phenotypes confer differential risks for diabetes-related complications. However, prospective data linking type 2 diabetes phenotypes to all-cause and cardiovascular disease mortality remain strikingly limited, particularly from South Asian populations. Similarly, defining heterogeneity in the early natural history of dysglycemia, i.e., prediabetes, is pivotal for precision diabetes prevention and identification of high-risk individuals. Thus far, rigorous identification of prediabetes subtypes and their association with long-term outcomes has been extremely limited.^16,17^

Understanding how type 2 diabetes and prediabetes subtypes influence clinical outcomes has profound implications for advancing precision medicine. Leveraging the CArdiometabolic Risk Reduction study cohort,^18–20^ a socio-demographically representative, prospective study of adults aged 20 years and older from Delhi and Chennai with comprehensive glycemic assessments and well-retained long-term follow-up,^21^ we sought to address three key aims: first, to identify subtypes of type 2 diabetes and prediabetes, we used clustering variables originally applied by Ahlqvist et al.;^7^ second, to evaluate the association of these subtypes with all-cause and cardiovascular disease mortality over a median follow-up of 10.6 years; and third, to estimate excess years of life lost associated with each subtype. By deriving subtypes using the same set of variables in both type 2 diabetes and prediabetes, we sought to understand the natural progression of metabolic dysfunction and its prognostic relevance. This study is the first, to our knowledge, to characterize both type 2 diabetes and prediabetes subtypes and their long-term mortality outcomes in a large, prospective South Asian cohort.

## METHODS

### CARRS Cohort.

This study is a retrospective analysis of a prospective cohort, the Centre for Cardiometabolic Risk Reduction in South Asia (CARRS) study (n = 21,862), designed to investigate the natural history of cardiometabolic disease in adults aged ≥ 20 years across urban sites in India. Detailed methods are provided elsewhere.^18–20^ Briefly, participants were recruited in two waves using identical multistage cluster sampling methods for representative household selection. The cohort has maintained excellent response and retention rates (up to 14 years of follow-up) with minimal attrition.^21^

In the first wave (CARRS-1), a probability sample of 12,271 adults aged ≥ 20 years was enrolled during 2010–2011, designed to be representative of the urban populations in Chennai and Delhi. Follow-up assessments were conducted in 2012–13 (1st), 2013–14 (2nd), 2014 (3rd), 2016–17 (4th), 2017–18 (5th), and 2020–2021 (6th). In the second wave (CARRS-2), an independent probability sample of 9,591 adults was recruited in 2014–2016 using the same sampling and measurement methodology, with follow-ups conducted in 2018–2020 (1st). The cohort was recently funded to extend follow-up and conduct deep subclinical and clinical phenotyping to advance precision medicine efforts in South Asians (Precision-CARRS; P01HL154996). The most recent follow-up for both waves is being conducted from January 2023 through December 2024 (7th and 2nd respectively), including deep vascular subtyping assessments as part of these newly funded activities.

The study was approved by the Ethics Committees of the Public Health Foundation of India (IRB00006658), All India Institute of Medical Sciences, New Delhi (IEC/NP-17/07.09.09), Madras Diabetes Research Foundation, Chennai (MDRF/EC/EPI/2009/10), and Emory University, Atlanta, USA (IRB00044159). The Centre for Chronic Disease Control received clearance from the Indian Health Ministry’s screening committee.

Centrally trained personnel administered standardized,^22,23^ in-person interviews to gather medical histories at each follow-up visit. Blood pressure was measured three times in the seated position after a five minute rest period using an automated oscillometric device (Automated Omron HEM- 7080 and HEM-7080IT-E; Omron Healthcare Co., Ltd., Kyoto, Japan; certified by the British Hypertensive Society and the American association for Advancement of Medical Instrumentation protocols), and the mean of the last two readings was used for analyses. Height was measured using a portable stadiometer (SECA Model 213, SecaGmbh Co, Hamburg, Germany), and weight was recorded with a body composition analyzer (Tanita BC-408). Body mass index (BMI) was calculated as weight (kg) divided by height squared (m^2^).

Biochemical analyses were performed in accredited laboratories^24^ in Delhi and Chennai using automated analyzers (Beckman Coulter AU 680, Fullerton, CA, USA) with appropriate quality control procedures. Venous blood samples were analyzed on the day of collection. Plasma glucose was measured in the fasting state and at 30 minutes and 2 hours as part of a 75-g oral glucose tolerance test using hexokinase/kinetic based methods. Total cholesterol and triglycerides were measured enzymatically; high-density lipoprotein (HDL) cholesterol was measured directly; and Low-density lipoprotein (LDL) cholesterol was calculated using the Friedewald formula. Glycated hemoglobin (HbA1c) was measured by high-performance liquid chromatography (Variant II Turbo, Bio-Rad, Hercules, CA, USA) and standardized according to the National Glycohemoglobin Standardization Program. Serum insulin was measured using an electrochemiluminescence immunoassay (COBAS E411 analyzer, Roche Diagnostics, Mannheim, Germany). Estimates of pancreatic beta-cell function (HOMA2-B) and insulin resistance (HOMA2-IR) were derived from fasting plasma glucose and insulin levels using the HOMA2 calculator (https://www.dtu.ox.ac.uk/homacalculator/).^25^

All assays underwent stringent internal and external standardization procedures. Intra assay & inter-assay coefficients of variation were < 3% & 5% respectively. At least two levels of internal controls were included with each assay run. Laboratories participated in External Quality Assurance Scheme by RANDOX, with absolute standard deviation index values < 1 and all parameters meeting predefined acceptability criteria (≤ 2). For insulin assays, the laboratories also participated in the UK National External Quality Assessment Service, demonstrating performance within acceptable limits (< 15% variance; threshold ≤ 25%).

### Definitions for Diabetes and Prediabetes.

Diabetes was defined as self-reported, physician-diagnosed diabetes and/or use of diabetes medication.^26^ Self-reported diabetes history was validated against medical records, which also provided the date of diagnosis and facilitated calculation of age at diagnosis. Newly diagnosed diabetes was defined as no prior diagnosis of type 2 diabetes, with fasting plasma glucose ≥ 126mg/dL and/or 2-hour post-challenge glucose ≥ 200mg/dL, and/or HbA1c ≥ 6.5%.^27^ Prediabetes was defined as fasting plasma glucose 100–125 mg/dL (impaired fasting glucose), and/or 2-hour post-challenge glucose 140–199 mg/dL (impaired glucose tolerance), and/or HbA1c 5.7–6.4%, in the absence of prior diabetes. Normal glucose tolerance was defined as no self-reported history of diabetes and fasting plasma glucose < 100 mg/dL, 2-hour post-challenge glucose < 140mg/dL, and HbA1c < 5.7%.

### Mortality Outcomes.

The outcomes were all-cause and cardiovascular disease mortality. Additional outcomes included premature all-cause and cardiovascular disease mortality, defined as death before 70 years of age. Through August 2024, the date of the most recent completed follow-up or death, participants completed up to seven follow-up visits. Retention rates ranged from 70–85% at each visit and > 95% of participants have completed at least one follow-up or vital status assessment.^28^

For deceased participants, the date of death was confirmed using medical records or death certificates. When documentation was unavailable, a verbal autopsy was conducted with the next-of-kin (contact information collected at baseline). Two independent study physicians reviewed all available records to classify endpoints according to prespecified criteria. Discrepancies were resolved by consensus in consultation with a third senior investigator. Cardiovascular disease deaths were classified based on death certificates, medical records, and next-of-kin interviews and included deaths from coronary heart disease and stroke.

### Statistical analysis

All statistical analyses were conducted using R software (version 4.4.1; R Foundation for Statistical Computing). The reporting of results adhered to the Strengthening the Reporting of Observational Studies in Epidemiology guidelines.^29^ The demographic characteristics of the analytic sample, including age, sex, occupation, and educational attainment, were broadly comparable to those of the larger CARRS cohort, which is representative of urban populations in Delhi and Chennai.^18–20^

### Classification of type 2 diabetes and prediabetes subtypes.

For this analysis, we excluded participants with missing baseline variables that were used for clustering using five well-established clinical variables: age, BMI, HbA1c, HOMA2-IR, and HOMA2-B in type 2 diabetes and prediabetes individuals. This data-driven approach was first introduced by Ahlqvist et al.,^7^ who applied an unsupervised k-means clustering algorithm to identify reproducible subtypes of type 2 diabetes. The framework has since been validated in > 20 studies globally.^14,15^ Given the distinct phenotypic characteristics and distribution of these variables in South Asian populations compared with European cohorts, we applied de novo clustering.

Clustering was restricted to participants with type 2 diabetes (n = 2,639) or prediabetes (n = 4,992); individuals with normal glucose tolerance (n = 6,405) were not included in the clustering process but were retained in the full analytic sample for downstream association analyses, resulting in a final clustering sample of 14,036 individuals. (ESM Figure-1). Clustering was performed separately for type 2 diabetes and prediabetes groups using the kmeansruns function from the fpc package,^30^ exploring solutions ranging from 2 to 10 clusters. The optimal number of clusters was identified based on the highest average silhouette width, selecting the most cohesive and distinct clusters. Cluster labels were assigned based on the mean phenotypic characteristics of each cluster.

Cluster robustness was evaluated using a permutation-based sensitivity analysis in which input variables were randomly shuffled across 100 iterations. Clustering was re-run on each permuted dataset to assess consistency of the original structure. A low adjusted Rand Index across permutations confirmed the stability of the observed clusters.

### Association Analysis.

Continuous variables were summarized as means with standard deviations or medians with interquartile ranges, and categorical variables as proportions. Median follow-up time, event counts, mortality rates per 1,000 person-years with 95% confidence intervals, and life expectancy estimates were reported, stratified by glycemic subtypes.

Associations between type 2 diabetes, prediabetes, and their respective subtypes with mortality outcomes were examined using Cox proportional hazards regression models that incorporated survey weights to account for the complex sampling design. Follow-up time was defined from baseline to death or the most recent follow-up. The proportional hazards assumption was evaluated using Schoenfeld residuals.^31^ Primary Cox proportional hazard models were adjusted for age (modeled using restricted cubic splines with 4 degrees of freedom), cohort (CARRS-1 | CARRS-2), city (Chennai | Delhi), sex (Male | Female), baseline levels of systolic blood pressure, total cholesterol levels, self-reported medical history of previous non-fatal cardiovascular disease events (Yes | No), and tobacco consumption (Yes | No). Survival differences between type 2 diabetes and prediabetes subtypes were assessed using pairwise comparisons, and hazard ratios were calculated to estimate relative risks. Premature all-cause and cardiovascular disease mortality were evaluated, using the same modeling strategy.

Associations of type 2 diabetes and prediabetes, defined using standard diagnostic criteria, with mortality outcomes were evaluated to compare their prognostic performance with the newly identified subtypes. Prognostic performance was assessed using log-likelihood, Akaike Information Criterion (AIC), and concordance analysis.

### Life Expectancy and Excess Years of Life Lost.

Life expectancy was estimated using the restricted mean survival time method, which reflects the average survival time from baseline to end of follow-up. 32 Restricted mean survival times were calculated for each type 2 diabetes and prediabetes subtype, as well as for individuals with normal glucose tolerance, using observed survival data, with corresponding 95% confidence intervals derived from 1,000 bootstrap replications. Excess life-years lost were defined as the difference in restricted mean survival time between each glycemic subtype and the normal glucose tolerance group and were estimated using lillies package in R.^32^

### Contribution of metabolic variables.

We examined the metabolic variables used for subtyping and examined their individual associations with all-cause mortality. For each cluster, standardized values of HOMA2-IR, BMI, HOMA2-B, and HbA1c were entered as covariates into separate Cox proportional hazards models, with all-cause mortality as the outcome. Hazard ratios with 95% confidence intervals were estimated to quantify the associations between each variable and mortality risk across subtypes.

## Results

### Study Population

The analytic cohort included 14,036 participants: 6,405 (45.6%) with normal glucose tolerance, 4,992 (35.6%) with prediabetes, and 2,639 (18.8%) with type 2 diabetes at baseline (ESM Figure-1). Of these, 6,497(46.3%) were male. Participants had a mean age of 42.1 ± 13.0 years, a BMI of 25.5 ± 5.1 kg/m^2^, an HbA 1 c of 5.9 ± 1.2%, a HOMA2-IR of 1.5 ± 1.0, and a HOMA2-B of 91.0 ± 42.0. Full baseline anthropometric, clinical, and laboratory characteristics, stratified according to standard American Diabetes Association–classifications for type 2 diabetes and prediabetes, are shown in ESM Table-1.

#### Distinct type 2 diabetes and Prediabetes Subtypes.

**Figure 1:**
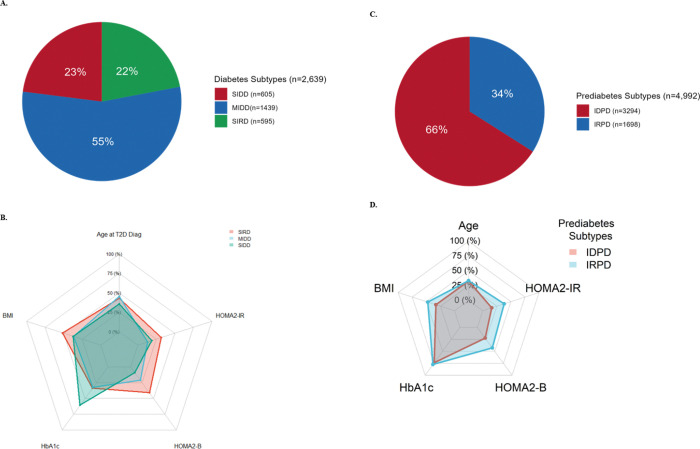
Characteristics of the Type 2 Diabetes and prediabetes data-driven subtypes in the CARRS cohort A. Type 2 Diabetes subtypes distribution; B. Type 2 Diabetes phenotypic characteristics; C. Prediabetes subtypes distribution; D. Prediabetes phenotypic characteristics. SIDD: severe insulin deficient diabetes; MIDD: mild insulin deficient diabetes phenotype; SIRD: severe insulin resistant diabetes; IDPD: insulin deficient prediabetes; IRPD: Insulin resistant prediabetes

Unsupervised clustering analysis identified three subtypes of type 2 diabetes and two subtypes of prediabetes (Figure-1A-B), with distinct phenotypic characteristics (Table-1). Permutation analysis established the statistical robustness of these clusters. Among individuals with type 2 diabetes, 77.5% exhibited features of insulin deficiency. The Severe Insulin-Deficient Diabetes (SIDD) subtype, comprising 23% of type 2 diabetes cases, was characterized by early-onset diabetes, pronounced β-cell dysfunction, and markedly elevated HbA1c levels. The Mild Insulin-Deficient Diabetes (MIDD) subtype, the most prevalent (54.5%), was associated with lower BMI, intermediate HbA1c levels, and reduced insulin secretion relative to SIRD. The remaining 22.5% had the Severe Insulin-Resistant Diabetes (SIRD) subtype, distinguished by elevated BMI, marked insulin resistance, increased insulin secretion, and relatively well-controlled HbA1c levels. SIDD was more common in males, whereas SIRD was more frequent in females (ESM Figure-2A).

In the prediabetes group, two subtypes were identified: the Insulin-Deficient Prediabetes (IDPD) phenotype, comprising 66%, and the Insulin-Resistant Prediabetes (IRPD) phenotype, comprising 34% (Figure-1C-D). The distribution of individuals classified by standard American Diabetes Association prediabetes categories was similar between IDPD and IRPD (ESM Fig. 3). IDPD was more frequent in males, while IRPD was more prevalent in females (ESM Fig. 2B).

#### Risk of All-Cause and Cardiovascular Mortality.

Over a median (interquartile range) follow-up of 10.6 years (IQR, 8.2–12.0; 140,640 person-years), 1,076 all-cause deaths occurred: 268 (4%) among individuals with normal glucose tolerance, 357 (7%) among those with prediabetes, and 451 (17%) among those with type 2 diabetes. Among type 2 diabetes subtypes, 149 (25%) deaths occurred in the SIDD group, 228 (16%) in the MIDD group, and 74 (12%) in the SIRD group (Table 2). Within prediabetes subtypes, 270 (8%) deaths occurred in the IDPD group, and 87(5%) in the IRPD group.

**Figure 2:**
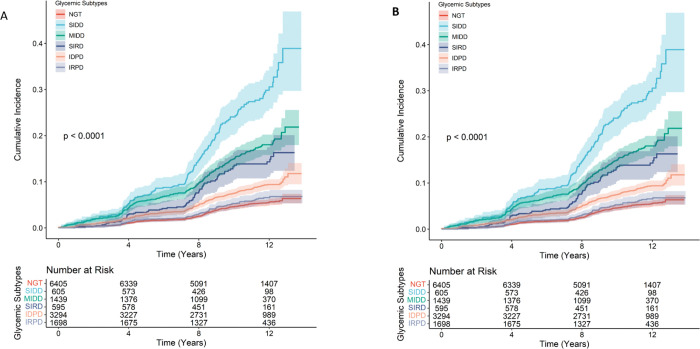
**A:** Cumulative Incidence of Overall Mortality by Type 2 Diabetes and Prediabetes Subtypes. **B:** Cumulative Incidence of CVD Mortality by Diabetes and Prediabetes Subtypes. SIDD: severe insulin deficient diabetes; MIDD: mild insulin deficient diabetes phenotype; SIRD: severe insulin resistant diabetes; IDPD: insulin deficient prediabetes; IRPD: Insulin resistant prediabetes.

A stepwise increase in the risk of all-cause and cardiovascular disease mortality was observed with worsening metabolic profiles, accompanied by substantial heterogeneity across glycemic subtypes (Figure-2A-B). Among individuals with normal glucose tolerance, the all-cause mortality rate was 4.3 (95% CI, 3.8 to 4.8) per 1000 person-years. In comparison, markedly higher rates were observed among T2D subtypes, particularly those characterized by insulin deficiency. The SIDD subtype had the highest mortality rate at 26.2 (95% CI, 22.1 to 30.7) per 1000 person-years, with an absolute rate difference of 21.9 (95% CI, 17.6 to 26.2) per 1000 person-years compared with normal glucose tolerance. MIDD and SIRD subtypes also showed elevated mortality rates with the death rates of 15.8 (95% CI, 13.8 to 18.0) per 1000 person-years and 12.4 (95% CI, 9.7 to 15.5) per 1000 person-years, respectively, with corresponding rate differences of 11.5 (95% CI, 9.3 to 13.7) per 1000 person-years and 8.1 (95% CI, 5.2 to 11.0) per 1000 person-years. Among prediabetes subtypes, IDPD exhibited a higher mortality rate of 7.8 (95% CI, 6.9 to 8.8) per 1000 person-years, with an absolute risk difference of 3.5 (95% CI, 2.4 to 4.6, reference normal glucose tolerance). In contrast, the IRPD subtype showed no meaningful excess risk (rate difference: 0.7 (95% CI, −0.5 to 1.9). Similar patterns were observed for associations between T2D and prediabetes subtypes and cardiovascular disease mortality (Table-2).

After adjustment for age, sex, cohort, city, tobacco use, medical history of cardiovascular disease events, systolic blood pressure, and total cholesterol, among type 2 diabetes subtypes, SIDD had the highest risk of all-cause mortality (adjusted HR: 3.34; 95% CI, 2.39 to 4.68) compared with normal glucose tolerance. MIDD (adjusted HR: 1.39; 95% CI, 1.05 to 1.84) and SIRD (adjusted HR: 1.67; 95% CI, 1.15 to 2.41) also showed increased risk, though less pronounced. Among prediabetes subtypes, IDPD conferred a 1.3-fold higher risk of all-cause mortality (adjusted HR: 1.32; 95% CI, 1.03 to 1.68) compared with normal glucose tolerance, while IRPD was not associated with higher mortality (adjusted HR: 0.84; 95% CI, 0.59 to 1.21).

Cardiovascular disease-related deaths occurred in 73 participants (1.1%) with normal glucose tolerance, 128 (4.9%) with prediabetes (93 [3.0%] in the IDPD group and 35 [2.0%] in the IRPD group), and 204 (7.7%) with type 2 diabetes (149 [14.2%] in SIDD, 80 [5.6%] in MIDD, and 38 [6.4%] in SIRD). In the fully adjusted models (Table-2), among type 2 diabetes subtypes, SIDD had the highest risk of cardiovascular disease mortality (HR: 5.94; 95% CI, 3.46 to 10.20) compared with normal glucose tolerance. The MIDD (HR: 1.62; 95% CI, 1.04 to 2.52) and SIRD (HR: 2.30; 95% CI, 1.35 to 3.91) subtypes were also associated with increased risk, though less pronounced than SIDD. Among prediabetes subtypes, IDPD was linked to a 1.5-fold higher cardiovascular disease mortality risk (HR: 1.53; 95% CI, 1.00 to 2.34), while IRPD showed no significant association (HR: 0.81; 95% CI, 0.47 to 1.38).

#### Risk of Premature All-Cause and Cardiovascular Mortality.

A total of 421 deaths (39.1%) were classified as pre-mature mortality. These included 145 deaths in the normal glucose tolerance (2.3%) group, 149 (3.1%) in the prediabetes group, and 127 (5.5%) in the type 2 diabetes group. Among those with type 2 diabetes, 56 (44% of T2D-related premature deaths) premature deaths occurred in the SIDD group, 50 (39%) in the MIDD group, and 21 (17.0%) in the SIRD group. Within prediabetes subtypes, 116 (78% of prediabetes-related deaths) deaths occurred in the IDPD group, and 33 (22%) in the IRPD group. In fully adjusted models (ESM Table-2), all three type 2 diabetes subtypes showed significant associations with premature all-cause mortality, with the highest risk observed in SIDD (HR: 5.62; 95% CI, 3.79–8.34), followed by SIRD (HR: 2.36; 95% CI, 1.50–3.70) and MIDD (HR: 1.87; 95% CI, 1.29–2.69). Among prediabetes subtype, IDPD was associated with increased risk (HR: 1.56; 95% CI, 1.17, 2.08), while IRPD was not. For premature cardiovascular disease mortality, SIDD (HR: 6.85; 95% CI, 3.65, 12.82) and SIRD subtype (HR: 2.26; 95% CI, 1.20, 4.25) showed significant associations. No prediabetes subtype was associated with premature cardiovascular disease mortality.

#### Excess Years of Life Lost.

The insulin-deficient subtypes accounted for the greatest estimated reductions in life expectancy for both all-cause and cardiovascular disease mortality (Table-2). Among individuals with type 2 diabetes, excess years of life lost relative to normal glucose tolerance were 17.7 years (95% CI, 17.1 to 18.1) for SIDD, 12.8 years (95% CI, 12.6 to 13.3) for MIDD, and 12.0 years (95% CI, 10.0 to 12.8) for SIRD. Compared to those with normal glucose tolerance, IDPD was associated with 4.8 years [95% CI, 4.6 to 5.1] excess years of life lost, and IRPD with 0.6 excess years of life lost [95% CI, − 0.5 to 1.4]. For cardiovascular disease mortality, only the SIDD was associated with a significant excess year of life lost (9.4; 95% CI, 6.4 to 12.2]), while the other subtypes did not show any appreciable excess.

#### The added value of clustering beyond traditional glycemic criteria and the biomarkers.

The data-driven subtypes of type 2 diabetes and prediabetes demonstrated stronger association with all-cause mortality than the standard ADA-defined glycemic categories (ESM Table-3). While standard classification of type 2 diabetes was associated with an increased mortality risk (HR: 1.87; 95% CI, 1.44 to 2.42]), the clustering approach revealed substantial heterogeneity within type 2 diabetes. In contrast, standard prediabetes criteria were not significantly associated with all-cause mortality (HR: 1.17; 95% CI, 0.93 to 1.48), whereas the IDPD subtype was significantly associated with increased mortality risk.

Model fit was significantly improved with the subtype classification compared to standard glycemic categories (*P* < 0.001 by likelihood ratio test). Concordance was also marginally higher for the subtype-based model (0.810 vs.0.803). The individual variables included in the model were also associated with the mortality outcomes. HbA1c was positively associated with all-cause mortality (HR: 1.30; 95% CI, 1.22 to 1.39), whereas BMI (HR: 0.80; 95% CI, 0.72 to 0.81) and HOMA2-B (HR: 0.72; 95% CI, 0.64 to 0.81) were inversely associated. HOMA2-IR showed no significant association with mortality (HR: 1.04; 95%CI, 0.95 to 1.14).

#### Relative contributions of individual biomarkers within clusters to all-cause mortality.

We next examined the relative contributions of these biomarkers within each type 2 diabetes and prediabetes subtype (Table-3). Among individuals with type 2 diabetes, BMI was inversely associated with mortality in both SIDD (HR: 0.80; 95% CI, 0.66 to 0.95) and MIDD (HR: 0.78; 95% CI, 0.64 to 0.84). HOMA2-B was the principal predictor in SIDD (HR: 0.70; 95% CI, 0.55 to 0.88), while HbA1c was the primary determinant in MIDD (HR: 1.23; 95% CI, 1.10 to 1.40). HOMA2-IR was not significantly associated with mortality in any of the type 2 diabetes subtypes. In prediabetes subtypes, BMI was inversely associated with all-cause mortality in IDPD (HR: 0.58; 95% CI, 0.50 to 0.66), while other biomarkers did not show significant associations.

## Discussion

In this population-based cohort from urban India, we applied validated, data-driven clustering to identify three distinct type 2 diabetes subtypes (SIDD, MIDD, and SIRD) and two novel prediabetes (IDPD and IRPD) subtypes, revealing substantial heterogeneity in mortality risk. Insulin-deficient subtypes predominated, accounting for 77.5% of all diabetes cases and 66.0% of prediabetes cases. Notably, SIDD subtype was associated with the highest risks of all-cause, cardiovascular disease, and premature mortality, as well as marked reductions in life expectancy. Collectively, insulin-deficient type 2 diabetes and prediabetes subtypes (SIDD, MIDD, and IDPD) accounted for 80.1% of diabetes-related deaths, contributing disproportionately to premature mortality. These findings underscore the central role of pancreatic beta-cell dysfunction in the pathogenesis of diabetes for South Asians, in contrast to insulin-resistant subtypes, which were associated with comparatively modest or no excess mortality risk. Stratification of individuals based on pathophysiologic subtypes, rather than relying solely on standard diabetes and prediabetes classifications, we reveal a more nuanced understanding of mortality risk, one that is often obscured by traditional clinical definitions.

Our findings, based on clustering algorithms incorporating five traits (age, BMI, HbA1c, HOMA2-IR, and HOMA2-B), revealed distinct type 2 diabetes subtypes compared to those reported in European and Scandinavian cohorts, despite using identical variables. Insulin-deficient subtypes were more prevalent in our cohort, reflecting unique metabolic and demographic characteristics of South Asians.^14,15^ These results align with prior studies highlighting type 2 diabetes heterogeneity in South Asians. Prasad et al.^4^ analyzed young-onset type 2 diabetes patients in India using the same clustering variables but with centroids derived from European populations. Insulin deficiency emerged as a predominant feature, yet de novo clustering identified only two groups: one primarily composed of SIDD (66%) and the other resembling Mild Obese Diabetes (MOD;34%). Conversely, the INSPIRED study by Anjana et al.,^3^ which incorporated additional metabolic traits (e.g., waist circumference, triglycerides, HDL cholesterol, and fasting and stimulated C-peptide), identified Mild Age Related Diabetes (MARD) as the predominant subtype (35.8%) in an Indian population, alongside SIDD (26%) and two other subtypes: insulin-resistant obese diabetes (IROD; 26%) and combined insulin-resistant and deficient diabetes (CIRDD; 12.1%). Congruently, pooled analyses of multiethnic, population-based studies from the MESA and MASALA cohorts demonstrated racial and ethnic differences in diabetes subtypes, with a higher prevalence of SIDD among South Asians, whereas MARD was the most common subtype in other racial groups.^33^

Our study’s unique contribution lies in examining both T2D and prediabetes subtypes, revealing a continuum of insulin deficiency from prediabetes to overt diabetes and offering novel insights into disease progression. Additionally, we provide robust data on mortality outcomes, including life expectancy reductions, establishing the clinical relevance of early identification and targeted interventions for high-risk groups to refine public health strategies for South Asian populations. In contrast to a smaller US-based study, which found no significant association between diabetes subtypes and all-cause mortality and linked only MARD to cardiovascular disease mortality,^34^ our findings demonstrate that insulin-deficient subtypes (MIDD and SIDD) were associated with markedly higher risks of all-cause and cardiovascular disease mortality, whereas SIRD exhibited only modest associations yet contributed disproportionately to excess years of life-years lost.

Among individuals with prediabetes, we identified an IDPD accounting for nearly two-thirds of cases. Although the IRPD and IDPD subtypes were pathophysiologically distinct, they were similarly distributed across conventional ADA-defined prediabetes diagnostic classification. This underscores the limitations of current glycemic-based classification systems in capturing the underlying heterogeneity of prediabetes. The marked variation observed across both prediabetes subtypes highlights the shortcomings of a one-size-fits-all approach.^35^

Data-driven subtyping improved stratification for all-cause and cardiovascular disease mortality compared with standard diabetes and prediabetes criteria, or individual biomarkers. Traditional prediabetes categories were not associated with mortality after full adjustment. In contrast, the prediabetes subtype, IDPD, showed a strong and independent association with both outcomes. This high-risk subgroup would likely have been overlooked using standard glycemic classifications alone.

Model fit, as assessed by the likelihood ratio test, was significantly better for the subtype-based classification than for the standard glycemic categories (*P* < 0.0001), and concordance and AIC values, while moderate, consistently favored the subtyping approach. Collectively, these findings demonstrate the superior prognostic value of pathophysiology-based clustering, particularly for identifying high-risk insulin-deficient phenotypes, compared to standard glycemic classifications. By integrating multidimensional metabolic patterns, this approach enhances interpretability, improves clinical utility, and holds promise for advancing precision diabetes medicine strategies.

Our clustering approach was not intended to outperform individual biomarkers but to integrate multiple dimensions of glycemic pathophysiology into clinically meaningful subtypes. Although HbA1c and HOMA2-B were each independently associated with mortality, they did not fully account for the heterogeneity revealed through clustering. By study design, we did not adjust for HbA1c or HOMA2-B in models evaluating subtype associations with mortality, as these variables formed the basis of subtype classification; including them would constitute overadjustment. Notably, mortality risk was not solely driven by glycemia. For example, the MIDD and IDPD subtypes, both characterized by modest HbA1c levels, were associated with elevated mortality, highlighting the prognostic relevance of insulin-deficient phenotypes independent of hyperglycemia. While glucotoxicity may influence HOMA2-B values, particularly in the SIDD cluster (mean HbA1c 10.4%), the identification of insulin-deficient subtypes across the glycemic spectrum, including among individuals with prediabetes, supports the presence of primary beta-cell defect in South Asians rather than secondary suppression alone.^36^ Thus, clustering provides added explanatory value by capturing pathophysiologic patterns that are obscured when examining individual variables in isolation.

We used HbA1c as the primary glycemic marker to align with prior diabetes subtyping studies.^14^ Although oral glucose tolerance test-based variables may offer finer resolution in the non-diabetic range as it was shown in the previous clustering studies among individuals with prediabetes,^16,17^ their limited clinical use reduces their relevance for broader implementation. HbA 1c remained independently associated with mortality risk, including in prediabetes.^37^ Moreover, our data-driven approach revealed biologically distinct subtypes that spanned conventional glycemic categories, underscoring heterogeneity not captured by standard definitions.

BMI showed an inverse association with mortality outcomes, consistent with prior studies in Indian populations.^38,39^ This contrasts with findings from Europe and North America, where higher BMI is linked to increased mortality. Our subtype classification, which shows that combinations of BMI with measures of glycemia are most predictive of mortality, may even reconcile these apparent contradictions. These divergent patterns suggest population-specific factors may drive the distinct relationship between BMI and mortality in South Asians. The contribution of clustering variables to mortality also varied by subtypes: HOMA-2B was the dominant predictor in SIDD, whereas HbA1c was the key determinant in MIRD. Collectively, these findings highlight the distinct pathophysiological mechanisms across subtypes and reinforce the limitations of conventional glycemic classifications.

Our study has several notable strengths. These include its prospective design, large sample size, extended duration of follow-up, and its focus on South Asians, a population that is both underrepresented in precision diabetes research^40,41^ and disproportionately affected by diabetes. The inclusion of individuals with prediabetes, along with long-term follow-up, allowed for a comprehensive life course assessment of baseline glycemic subtypes continuum and their relationship to mortality risk. A key strength lies in the ability to robustly evaluate insulin-deficient subtypes, particularly given their higher prevalence in this population, even at the prediabetes stage. This enabled more precise estimation of hazard ratios. In contrast to prior studies that have largely focused on European populations, where SIDD is relatively rare and phenotypes such as SIRD, MOD, and MARD are more common, our study provides critical insight into subtype-specific risks in a distinct and high-risk ethnic group.^15^ Furthermore, the rigorous and validated definitions of glycemic status and clinical outcomes enhance the reliability and reproducibility of our findings.

The study population was drawn from two major urban centers in South Asia, which may limit the generalizability of our findings to rural populations or other regional contexts who generally exhibit lower levels of metabolic morbidities and higher mortality following diabetes. Whether the subtypes identified in our study present similarly in these settings should be explored in future investigations. However, recent studies have noted a higher prevalence of insulin-deficient phenotypes among minority populations, such as African Americans, suggesting that our findings may have broader global relevance.^33,42^ Furthermore, prior studies have shown that individuals classified as having SIDD may transition to milder insulin-deficient subtypes over time.^43^ Consistent with this, we also identified milder forms of insulin-deficient diabetes (MIDD) in our cohort. These findings underscore the dynamic nature of diabetes phenotypes. In our study, subtypes were defined at baseline using age, BMI, HbA1c, insulin resistance, and beta-cell function. Because insulin measurements were not repeated during follow-up, we were unable to assess the stability of subtype classifications over time. Although prior studies with similar follow-up durations have shown cluster stability over 8 years with an accuracy of 59–72%,^43^ this represents a limitation but also highlights the evolving topical interest in evaluating type 2 diabetes and prediabetes heterogeneity and the importance of repeated assessments in future studies.

In future studies, the stratification of type 2 diabetes and prediabetes subtypes could be further refined with the integration of additional variables, including advanced biomarkers, polygenic risk scores, and proteomic data. Future multiomic studies are needed to elucidate the molecular basis of these subtypes and to provide deeper insights into their etiological mechanisms.^44^ More importantly, we need evidence from Mendelian Randomization techniques to support the value of phenotypic clustering in risk profiling. Finally, the clinical utility of the identified subtypes requires further investigation to determine how these phenotypic distinctions can be translated into clinical practice, improving personalized treatment strategies and diabetes management outcomes.

In conclusion, our findings reveal substantial heterogeneity in type 2 diabetes and prediabetes subtypes with disproportionately high prevalence of insulin-deficient subtypes. Moreover, the relative mortality risk as well as the absolute numbers of life years lost were highest among those exhibiting an insulin-deficient subtype, highlighting their major toll on mortality and life years lost in South Asians. These results suggest that standard classifications of type 2 diabetes and prediabetes may fail to capture the underlying pathophysiology, emphasizing the need for more nuanced frameworks to improve risk stratification and inform precision diabetes care.

## Supplementary Material

Supplementary Files

This is a list of supplementary fi les associated with this preprint. Click to download.

• CARRST2DPrediabetessubtypesFINALESM.docx

## Figures and Tables

**Table-1:** Baseline Characteristics of Participants in the CARRS Cohort by Diabetes and Prediabetes Subtypes.

	Normal glucose tolerance (n=6405)	Type 2 Diabetes (n=2639)			Prediabetes (n=4992)	
	
		SIDD (n=605)	MIDD (n=1439)	SIRD (n=595)	IDPD (n=3294)	IRPD (n=1698)

Age — Yr	37.2 (11.5)	49.8 (11.3)	52.6 (12.5)	50.4 (11.1)	43.4 (12.7)	43.8 (11.8)

Female sex — no. (%)	3391 (52.9)	272 (45.0)	746 (51.8)	356 (59.8)	1781 (54.1)	993 (58.5)

BMI —Kg/m^2^*	23.9 (4.6)	27.1 (4.8)	26.8 (4.2)	32.5 (5.2)	24.2 (4.1)	29.6 (4.5)

Blood pressure — mmHg						
Systolic	118.6 (17.4)	132.8 (21.1)	131.8 (20.7)	130.8 (19.0)	122.4 (18.8)	125.6 (18.5)
Diastolic	84.7 (11.1)	86.1 (11.7)	85.1 (12.0)	87.0 (11.3)	81.3 (11.4)	78.0 (11.3)

Self-reported tobacco consumption — no. (%)	1502 (23.5)	153 (25.3)	344 (23.9)	105 (17.6)	852 (25.9)	299 (17.6)

Family history of T2D — no. (%)	1511 (23.6)	199 (32.9)	384 (26.7)	185 (31.1)	646 (19.6)	419 (24.7)

Self-reported T2D — no. (%)						
Age at diabetes diagnosis, Years*	NA	379 (62.6)	489 (34.0)	196 (32.9)	NA	NA
	NA	43.3 (10.9)	50.1 (11.9)	48.2 (10.7)	NA	NA

Self-reported CVD — no. (%)	60 (0.9)	41 (6.8)	107 (7.4)	39 (6.6)	79 (2.4)	40 (2.4)

Fasting plasma glucose — mg/dl	89.1 (6.4)	226.3 (67.9)	125.6 (27.8)	124.8 (34.7)	99.4 (9.0)	100.0 (9.4)

HbA1c — %*	5.3 (0.3)	10.4 (1.5)	6.9 (0.8)	7.0 (0.9)	5.8 (0.4)	5.9 (0.3)

Lipid profile — mg/dl						
Total cholesterol	171.2 (35.4)	193.8 (40.9)	188.9 (41.9)	186.9 (41.6)	181.2 (39.2)	186.7 (36.6)
Triglycerides	122.0 (81.2)	212.5 (160.6)	164.7 (107.6)	174.0 (91.6)	138.5 (92.6)	160.5 (91.2)
HDL-Cholesterol	43.7 (11.3)	42.18 (11.2)	44.5 (12.6)	42.0 (11.1)	45.3 (12.1)	41.7 (9.6)

Surrogate insulin measures						
HOMA2-B*	97.3 (40.4)	29.9 (21.2)	66.8 (26.2)	127.9 (45.8)	75.6 (20.9)	135.9 (34.4)
HOMA2-IR*	1.5 (0.6)	2.2 (1.2)	1.5 (0.6)	3.6 (1.8)	1.0 (0.4)	2.3 (0.8)

SIDD: Severe Insulin Deficient Diabetes; MIDD: Mild Insulin Deficient Diabetes; SIRD: Severe Insulin Resistant Diabetes; IDPD: Insulin Deficient Prediabetes; IRPD: Insulin Resistant Prediabetes

* variables used for clustering.

**Table-2:** Risk of All-Cause and CVD mortality by Type 2 Diabetes and Prediabetes Subtypes.

Outcome	NGT N=6405	Type 2 Diabetes (n=2639)			Prediabetes (n=4992)	
		SIDD N=605	MIDD N=1439	SIRD N=595	IDPD N=3294	IRPD N=1698
**All-cause mortality**						
Mortality /PYs at risk	268/62878	149/5690	228/14398	74/5978	270/34486	87/17210
Mortality rate (per 1000 person-years)	4.3 (3.8; 4.8)	26.2 (22.1; 30.7)	15.8 (13.8; 18.0)	12.4 (9.7; 15.5)	7.8 (6.9; 8.8)	5.0 (4.0; 6.2)
Rate difference (95% CI)	0 (Ref)	21.9 (17.6; 26.2)	11.5 (9.3; 13.7)	8.1 (5.2; 11.0)	3.5 (2.4; 4.6)	0.7 (−0.5; 1.9)
HR [95% CI]^^a^	Ref	3.21 [2.33, 4.41]	1.33 [1.03, 1.71]	1.53 [1.08, 2.18]	1.28 [1.02, 1.62]	0.78 [0.54, 1.11]
HR [95% CI]^^b^	Ref	3.34 [2.39, 4.68]	1.39 [1.05, 1.84]	1.67 [1.15, 2.41]	1.32 [1.03, 1.68]	0.84 [0.59, 1.21]
Excess YLL	Ref	17.7 [17.1; 18.1]	12.8 [12.6; 13.3]	12.0 [10.0; 12.8]	4.8 [4.6; 5.1]	0.6 [−0.5; 1.4]
**CVD mortality**						
CVD mortality/PYs at risk	73/62878	86/5690	80/14398	38/5978	93/34486	35/17210
CVD Mortality rate (per 1000 person-years)	1.2 (0.9; 1.5)	15.1 (12.1; 18.7)	5.6 (4.4; 6.9)	6.4 (4.5; 8.7)	2.7 (2.2; 3.3)	2.0 (1.4; 2.8)
Rate difference (95% CI)	0 (Ref)	13.9 (10.6; 17.2)	4.4 (3.1; 5.7)	5.2 (3.1; 7.3)	1.5 (0.9; 2.1)	0.8 (0.04; 1.6)
HR [95% CI]^^a^	Ref	6.87 [4.21, 11.20]	1.77 [1.18, 2.66]	2.53 [1.53, 4.18]	1.61 [1.08, 2.41]	0.87 [0.51, 1.47]
HR [95% CI]^^b^	Ref	5.94 [3.46, 10.20]	1.62 [1.04, 2.52]	2.30 [1.35, 3.91]	1.53 [1.00, 2.34]	0.81 [0.47, 1.38]
Excess YLL	Ref	9.4 [6.4; 12.2]	0.7 [−1.4; 2.7]	4.3 [0.0; 7.1]	1.3 [−0.8; 3.4]	2.0 [−1.3; 5.3]

^ Adjusted for: a. age (modeled using restricted cubic splines with 4 degrees of freedom), sex (Male | Female); b. further adjusted for cohort (CARRS1 | CARRS2), city (Delhi | Chennai), tobacco consumption (Yes | No), CVD medical history (Yes | No), systolic blood pressure, and total cholesterol at baseline.

SIDD: Severe Insulin Deficient Diabetes; MIDD: Mild Insulin Deficient Diabetes; SIRD: Severe Insulin Resistant Diabetes; IDPD: Insulin Deficient Prediabetes; IRPD: Insulin Resistant Prediabetes; HR: hazard ratio; PY: Person-Years; YLL: years life lost.

**Table-3:** Association of each subtype biomarker with all-cause mortality by diabetes and prediabetes subtypes

Subtypes	HR [95% CI]

**NGT**	
- BMI	0.80 [0.80; 0.98]
- HbA1c	1.06 [0.90; 1.25]
- HOM2IR	0.90 [0.58; 1.37]
- HOM2B	0.93 [0.63; 1.37]

**SIDD**	
- BMI	0.80 [0.66; 0.95]
- HbA1c	1.16 [0.98; 1.37]
- HOM2IR	1.11 [0.97; 1.28]
- HOM2B	0.70 [0.55; 0.88]

**MIDD**	
- BMI	0.78 [0.64; 0.84]
- HbA1c	1.23 [1.10; 1.40]
- HOM2IR	0.90 [0.77; 1.05]
- HOM2B	0.91 [0.78; 1.05]

**SIRD**	
- BMI	1.03 [0.80; 1.32]
- HbA1c	1.14 [0.89; 1.46]
- HOM2IR	1.20 [0.93; 1.52]
- HOM2B	0.84 [0.64; 1.11]

**IDPD**	
- BMI	0.58 [0.50; 0.66]
- HbA1c	1.14 [0.98; 1.27]
- HOM2IR	0.85 [0.71;1.02]
- HOM2B	1.05 [0.89;1.24]

**IRPD**	
- BMI	1.01 [0.81; 1.25]
- HbA1c	1.08 [0.87;1.35]
- HOM2IR	1.07 [0.82; 1.40]
- HOM2B	1.12 [0.85; 1.47]

NGT: Normal Glucose Tolerance; SIDD: severe insulin deficient diabetes; MIDD: mild insulin deficient diabetes phenotype; SIRD: severe insulin resistant diabetes; IDPD: insulin deficient prediabetes; IRPD: Insulin resistant prediabetes

## Data Availability

Data supporting this study’s findings are available to the corresponding author (JR: ram.jagannathan@emory.edu) upon reasonable request. The R codes and the R notebook for the reproducible analysis is available to the interested readers by contacting: ram.jagannathan@emory.edu.
